# Targeted next generation sequencing identified clinically actionable mutations in patients with esophageal sarcomatoid carcinoma

**DOI:** 10.1186/s12885-018-4159-2

**Published:** 2018-03-05

**Authors:** Hongyang Lu, Shifeng Yang, Huineng Zhu, Xiaoling Tong, Fajun Xie, Jing Qin, Na Han, Xue Wu, Yun Fan, Yang W. Shao, Weimin Mao

**Affiliations:** 1 Zhejiang Key Laboratory of Diagnosis & Treatment Technology on Thoracic Oncology (lung and esophagus), Zhejiang Cancer Hospital, NO.1 East Banshan Road, Gongshu District, Hangzhou, 310022 People’s Republic of China; 2 Department of Thoracic Medical Oncology, Zhejiang Cancer Hospital, Hangzhou, 310022 People’s Republic of China; 3 Department of Pathology, Zhejiang Cancer Hospital, Hangzhou, 310022 People’s Republic of China; 4 Translational Medicine Research Institute, Geneseeq Technology Inc, Suite 300, MaRS Centre, South Tower, 101 College Street, Toronto, ON M5G 1L7 Canada

**Keywords:** Esophageal sarcomatoid carcinoma, Next generation sequencing, Mutation profiling, Targeted therapy

## Abstract

**Background:**

Esophageal sarcomatoid carcinoma (ESC) is a rare disease with a mixture of both carcinomatous and sarcomatous components in the tumor. Its genetic background and mechanisms of oncogenesis remain largely unknown.

**Methods:**

Here we performed targeted next generation sequencing (NGS) on a pan-cancer gene panel in 15 ESC tumors to explore their genetic alterations, and aimed to identify clinically actionable mutations for future treatment instructions.

**Results:**

*TP53* alterations were identified in all patients. Alterations in receptor tyrosine kinases (RTK) were identified in 10 out of 15 patients. Members of downstream RAS and PI3-kinase pathways are also mutated in 10 patients, and *PIK3CA* is the top mutated gene in these pathways. In addition, we identified mutations on histone modification genes in 5 patients, including histone acetyltransferase gene *EP300* and its homologue *CREBBP*, lysine methyltransferase genes *KMT2A* and *KMT2B*, and lysine demethylase gene *KDM5A*. Finally, mismatch repair (MMR) genes and proofreading gene *POLE* all together were mutated in one third of the ESC patients.

**Conclusions:**

This is the first study to unravel the mutational profile of ESC tumors. Our findings could match 9 patients to the targeted therapies currently available in clinical practice or in active clinical trials, suggesting the potential utility of targeted therapies for this rare disease in the future.

**Electronic supplementary material:**

The online version of this article (10.1186/s12885-018-4159-2) contains supplementary material, which is available to authorized users.

## Background

Esophageal sarcomatoid carcinoma (ESC) is a rare malignant tumor with a reported incidence of below 2% in esophageal carcinomas [1, 2]. The majority of the patients were male at the age of 60 and the correlation with smoking behavior is not clear. The histological pattern of sarcomatoid carcinoma is characterized by a biphasic appearance of both carcinomatous and sarcomatous components, most frequently a mixture of the malignant spindle cells and squamous cells [3]. Molecular characterization now supports the theory that these two components are originated from the same tissue precursor rather than two independent precursors [4, 5]. For diagnostic pathology, two types of immunohistochemical biomarkers, including epithelial biomarkers (e.g. cytokeratin and epithelial membrane antigen) and mesenchymal biomarkers (e.g. vimentin, smooth muscle actin and S100) are used to identify the carcinomatous and sarcomatous components, respectively [6]. However, the etiology of this rare disease is unclear due to the lack of genetic information.

Previous reports of ESC are often sporadic case studies without in-depth investigation of genetic characteristics. As a result, surgery to dissect tumors and nearby lymph nodes, complemented with adjuvant chemotherapy and/or radiotherapy before or afterwards, is the most adoptive treatment to this disease, and targeted therapies still remain unavailable. The 3-year survival rate of ESC is close to 60%, which is much higher than that of esophageal squamous cell carcinoma (ESCC) [6, 7]. However, its 5-year survival rate is close to that of ESCC, likely due to a lack of effective treatments to recurrent diseases.

To explore the genomic features of ESC and provide evidences for developing targeted therapies, we performed targeted next generation sequencing (NGS) with a customized NGS panel, which covers exons of 416 cancer-relevant genes and introns of 16 fusions genes, to interrogate genomic alterations in 15 dissected primary ESC tumors. All tumor samples were sampled at the time of diagnosis through esophagoscopy, or from surgical dissections of tumor. Due to the lack of normal controls for these tissue samples, public and in-house databases were used to filter out germline mutations and identify somatic mutations. The sequencing results not only depicted multiple molecular pathways that were commonly influenced in this disease, but also revealed the genetic heterogeneity between individuals. Moreover, clinically actionable mutations were identified in the majority of patients, suggesting the potential of involving targeted drugs into future treatment to improve the long-term survival rates of this disease.

## Methods

### Sample collection and sequencing library preparation

Formalin-fixed paraffin-embedded (FFPE) blocks from 15 ESC patients between 2009 and 2016 were retrospectively collected from Zhejiang Cancer Hospital in China. Tumor tissues were taken either at the time of diagnosis through esophagoscopy, or from surgically removed tumors. Only patient #3 received neoadjuvant therapy (2 cycles of docetaxel anhydrous and cisplatin-based therapy) prior sample acquisition. FFPE sections containing both carcinoma and sarcoma components of the tumor were collected for genomic DNA extraction using QIAamp FFPE DNA kit (Qiagen, Hilden, Germany) according to manufacturer’s protocol. We were unable to collect paired normal DNA controls for each patient due to the death of the patients or their release from the hospital. All DNA was qualified using Nanodrop 2000 and quantified using Qubit 2.0 fluorometer with Qubit dsDNA HS Assay Kit (Thermo Fisher, Waltham, MA).

500 ng~ 1 μg of extracted DNA for each sample was sheared into 350 bp fragments using Covaris M220 instrument (Covaris, Woburn, MA), followed by library preparation using KAPA Hyper DNA Library Prep Kit (KAPA Biosystems, Wilmington, MA). In brief, fragmented DNA underwent sequential steps of end-repairing, A-tailing and ligation with indexed adapters, followed by size selection with Agencourt AMPure XP beads (Beckman Coulter, Mississauga, Canada) and PCR amplification.

A customized NGS panel targeting exons of 416 cancer-relevant genes and introns of 16 fusions genes was used for hybridization enrichment (Additional file 1: Table S1). In brief, indexed DNA libraries were pooled together to a total amount of 2 μg and subjected to probe-based hybridization using IDT xGen Lockdown reagents (IDT, Coralville, IA) and Dynabeads M-270 (Thermo Fisher). Captured libraries were on-beads amplified with Illumina p5 and p7 primers in KAPA HiFi HotStart ReadyMix (KAPA Biosystems). The final library was quantified by KAPA Library Quantification kit (KAPA Biosystems) per manufacturer’s instructions. Bioanalyzer 2100 (Agilent, Stanta Clara, CA) was used to determine the fragment size distribution of the final library.

### Sequencing and data processing

FFPE samples of dissected primary tumors were sequenced to an average sequencing coverage of at least 300× on Illumina Hiseq 4000 platform (Illumina, San Diego, CA). The sequencing data were first demultiplexed by bck2fastq and then subjected to Trimmomatic [8] for FASTQ file quality control (QC). Leading/trailing low quality (base phred score below 15) or N bases were removed. Qualified reads were mapped to the reference human genome hg19 using Burrows-Wheller Aligner (BWA-mem, v0.7.12) [9]. Genome Analysis Toolkit (GATK 3.4.0) was employed to apply the local realignment around indels and base quality score recalibration. PCR duplicates were removed by Picard (available at: https://broadinstitute.github.io/picard/). VarScan2 was employed for the detection of single-nucleotide variations (SNVs) and insertion/deletion mutations with the following parameters: minimum read depth = 20, minimum base quality = 15, minimum variant allele frequency (VAF) = 0.01, minimum variant supporting reads = 5, variant supporting reads mapped to both strands, and strand bias no greater than 10% [10]. ADTEx (http://adtex.sourceforge.net) was used to identify copy number variations (CNVs) using a normal human HapMap DNA sample NA18535, and the cutoff of log2 ratio was set at ±0.6 for copy number changes (corresponding to 1.5-fold copy number gain and 0.65-fold copy number loss).

### Variant filtering and annotation

The vcf files contain both single-nucleotide polymorphism (SNPs) and small insertions/deletions (indels) were annotated by ANNOVAR against the following databases: dbSNP (v138), 1000Genome, ExAC, COSMIC (v70), ClinVAR, and SIFT. Only missense, stopgain, frameshift and non-frameshift indel mutations were kept. Mutations were removed if they were present in > 1% population frequency in the 1000 Genomes Project or 65,000 exomes project (ExAC). The resulted mutation lists were filtered through an internally collected list of recurrent sequencing errors on the same sequencing platform, which is summarized from the sequencing results of 53 normal samples with a minimum average sequencing depth of 700×. Specifically, if a variant was detected (i.e. ≥3 mutant reads and > 1% VAF) in > 20% of the normal samples, it was considered a likely artifact and was removed. Mutations occurred within the repeat masked regions were also removed. In a further filtering step, the mutation was only called out when the VAF is above 1% with a minimum of 5 mutant reads for COSMIC mutations, or above 2% with a minimum of 8 mutant reads for non-COSMIC mutations.

## Results

### Patient information

Of the 15 ESC patients enrolled, 12 were males and 9 of them were heavy smokers (≥ 20 packs/year). All 3 female patients have no smoking history. The median age of all patients is 65, ranging from 46 to 70. Eleven patients were diagnosed with stage I-II diseases, and 4 patients had stage III diseases (Table 1). Primary tumors occurred mostly in the bottom two thirds of esophagus (14 patients in total) and 10 patients were classified as the polypoid type when inspected by esophagoscopy.

**Table 1:** Clinical characteristics of patients

Age at diagnosis (*n* = 15)	Years
Median	65
Range	46 - 70
Gender	No. of patients
Female	3
Male	12
Clinical stage	No. of patients
I	4
II	7
III	4
Primary tumor position in esophagus	No. of patients
Upper thoracic	1
Mid-thoracic	7
Lower thoracic	7
Macroscopic type	No. of patients
Polypoid type	10
Ulcerating type	1
Infiltrative type	4

### Molecular pathways that were influenced by identified mutations

**Fig. 1:**
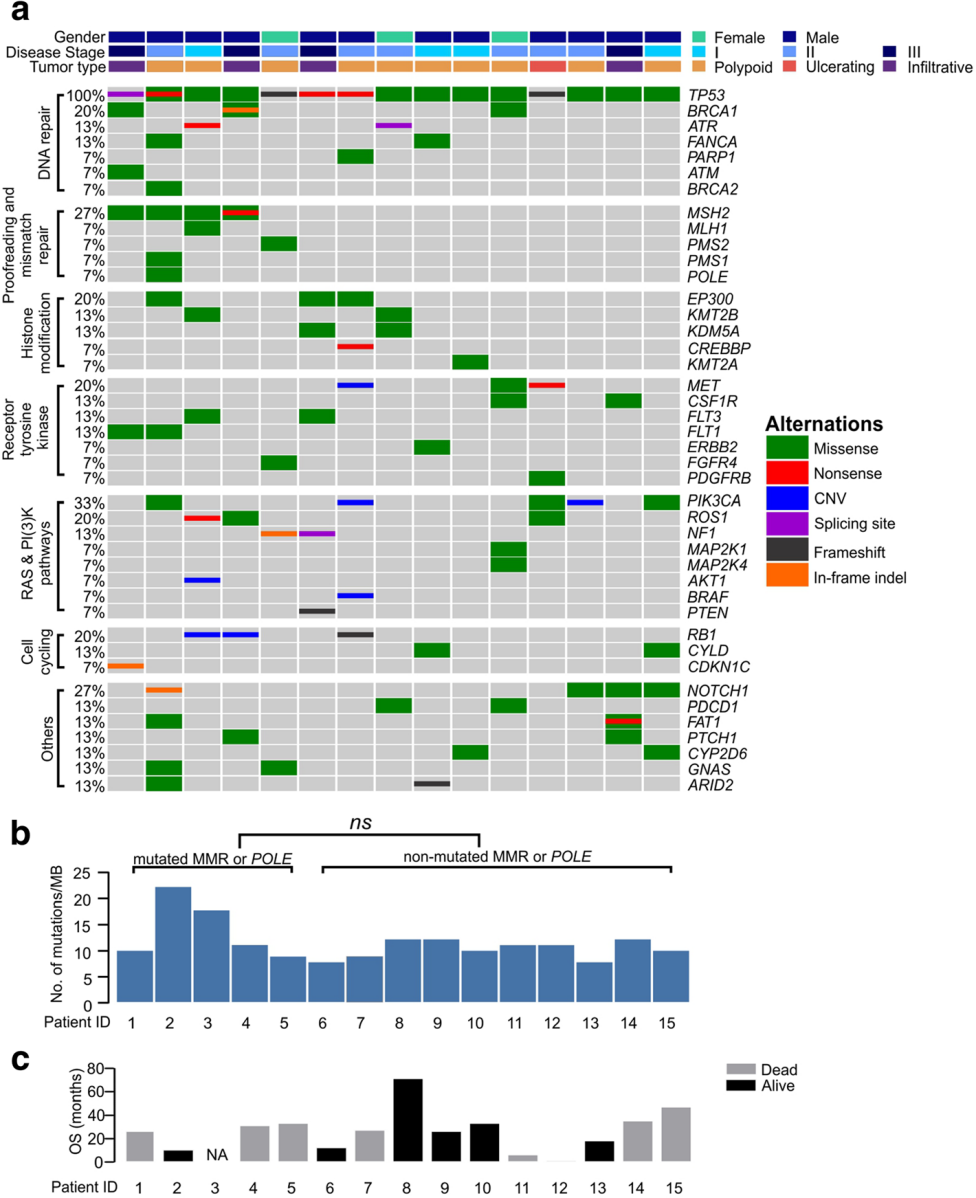
Mutation analysis of ESC patients. **a** A co-mutation plot of various types of mutations in all patients. Genes were grouped according to their functions. Each column represents one patient. The mutation rates of each gene were marked on the left in percentage and grouped according to their protein functions. Patient characteristics such as gender, disease stage and tumor type were shown at the top with different colors. **b** TMB in each patient. “ns”, not significant (Student’s t test, *p* < 0.05). **c** For overall survival (OS) time, black bars indicate that the patient was still alive at the time of last visit and grey bars indicate patients that were passed away. One patient was lost to follow-up and his OS time was marked as “NA”. All patients were placed in the same order in the 3 panels

Of these 15 patients, a total of 161 genetic alterations were identified in 94 genes (Additional file 2: Table S2). Six cases have mutations in histone modification genes. In particular, *EP300* was mutated in 3 cases and its homolog *CREBBP* in 1 case (Fig. 1a). Both proteins encoded by *EP300* and *CREBBP* are suggested as tumor suppressors and indicative of poor prognosis in ESCC [11]. All *EP300* mutations are in its histone acetyltransferase (HAT) domain and the mutation Y1414C which has already been reported as an inactivating mutation occurred in two patients [11]. In addition, 4 cases have mutations in *KMT2B*, *KDM5A* or *KMT2A*, all of which are components of histone methyltransferase complex (HMT). These results suggest that epigenetic regulation may play a critical role in ESC oncogenesis.

We also identified that receptor tyrosine kinases (RTK) and their downstream pathways were frequently mutated in these patients (Fig. 1a). Eleven out of 15 patients have at least one nonsynonymous mutation or structural variation in RTKs, including *MET, CSF1R, FLT3, FLT1*, *ETBB2, FGFR4 and PDGFRB*, and their occurrences were mutually exclusive from each other. As to the downstream elements of RTKs, *PIK3CA* is altered in 5 cases and 2 of them are copy number gain. The other 3 variants are E545K, M1043 V and H1047L, all of which have been reported to increase the catalytic activity of the p110α subunit of PIK3CA [12, 13].

Genes related to cell cycling and DNA repair is another commonly mutated category in ESC, but the mutation spectrum was not entirely the same as in ESCC. The mutation rate of *TP53* was quite similar between ESCC (93%) [11] and ESC (100%) (Fig. 1a), but much higher than the reported 73% in gynaecological carcinosarcoma, which is also a mixture of both carcinomatous and sarcomatous components with gynaecological origin [14]. Meanwhile, the loss of *RB1* gene was observed in 3 cases (20%) either by copy number loss or frameshift alteration, which is higher than its prevalence in ESCC (9%) [11]. We also observed that *CCND1* and *CDKN2A*, encoding two key molecules for cell cycling, were rarely altered in ESC (Additional file 2: Table S2), while *CCND1* amplification and *CDKN2A* mutations were found in 30% and 20% of ESCC patients, respectively [15].

The NOTCH pathway is also frequently dysregulated in ESC patients as we identified four *NOTCH1* mutations (27%) (Fig. 1a) and one *FBXW7* inactive mutation H460R (Additional file 1: Table S1) [16]. *NOTCH1* encodes a ligand-activated transcription factor in regulating cell differentiation, proliferation and apoptosis [17]. Mutations of *NOTCH1* are commonly identified in ESCC (13%) [11], acute and chronic lymphoblastic leukemia and suggested as oncogenic [18]. On the other hand, FBXW7 is a tumor suppressor and its inactivation could result in the constitutive activation of NOTCH1, cyclin E, c-Myc and other oncogenic factors [19, 20].

The tumor mutation burden (TMB) is defined as the number of non-synonymous and indel mutations per mega base (Mb) and it is ranged from 7.8 to 22.2 with a median of 11.1 in these patients (Fig. 1b). Notably, one third of the patients have either missense mutations or truncations in the mismatch repair (MMR) genes (*MSH2*, *MLH1*, *PMS2*, *PMS1*) and DNA polymerase *POLE* (Fig. 1a), which are all related to genomic stability [21, 22]. During variant annotation in ANNOVAR, SIFT tool was used to predict the influence of these mutations to their protein functions and ClinVar was used for the clinical significance annotation. We found that half of these mutations (patient #3, 4, 5) were predicted to be neutral in SIFT, and ClinVar only identified *MSH2* R929X (patient #3) as a pathogenic variant (Table 2). Correspondingly, TMB of these cases is not significantly higher than those without mutations in these genes (Fig. 1b). Patient #1 harbored one predicted-deleterious *MSH2* mutation, but demonstrated similar TMB as others. Only patient #2, whom was predicted to have functionally deleterious mutations in *PMS1*, *MSH2* and *POLE*, had a TMB of 22 mutations per MB, the highest among all studied cases.

**Table 2:** SNVs in MMR and proofreading genes

Patient ID	Gene	Mutation	SIFT	CLIN_SIG
1	*MSH2*	p.V78I (c.G232A)	Deleterious	–
2	*POLE*	p.E991Q (c.G2971C)	Deleterious	–
	*PMS1*	p.A6V (c.C17T)	Deleterious	–
	*MSH2*	p.A600T (c.G1798A)	Deleterious	–
3	*MSH2*	p.Q629R (c.A1886G)	Neutral	Not_provided;benign
	*MLH1*	p.Q701K (c.C2101A)	Neutral	Likely_benign;pathogenic
4	*MSH2*	p.R929X (c.C2785T)	–	Pathogenic
	*MSH2*	p.T564A (c.A1690G)	Neutral	Benign
5	*PMS2*	p.H435Q (c.C1305G)	Neutral	–

*CLIN_SIG:* clinical significance predicted by ClinVar

### Identification of clinically actionable mutations

Due to the poor understanding of ESC genomic profiles, currently there are no targeted therapies approved for these patients. In this study, patients were subjected to surgical desection and/or chemo/radiotherapies and their survival time was ranged from 1 month to exceeding 71 months (alive at the time of testing) after diagnosis (Fig. 1c), suggesting that their genetic background could possibly be related to the extraordinary differences of their prognosis. In this study, clinically actionable mutations were defined as mutations showing sensitivity to existing targeted therapies or drugs in clinical trials, or having been approved to influence the outcomes of targeted therapies, regardless of the cancer types in the original trials [23]. Of 15 ESC patients, 9 have been identified with at least one clinical actionable mutation based on the results of preclinical or clinical trials (Table 3) [24–32]. Taking *TP53* for example, supportive evidences include: 1) patients with carcinomas demonstrated better responses to bevacizumab treatment if carrying *TP53* mutations [24]; 2) patients with sarcomas showed better responses to pazopanib treatment [25]. In addition, the PI3-kinase pathway was activated in 6 patients by altering AKT1, PIK3CA or PTEN functions, and it can be targeted by multiple PI3K/mTOR/AKT or MEK inhibitors that are currently in active clinical trials [26, 27].

**Table 3:** Clinically actionable gene alterations

Gene	Alterations	Patient ID	Significances in treatment and prognosis
*AKT1*	Amplification	3	Response to mTOR inhibitors, AKT inhibitor MK2206 [28]
*CREBBP*	p.Q540X (c.C1618T)	7	Response to HDAC inhibitors (active clinical trial) [29]
*HNF1A*	p.288 fs (c.864_865insC)	3	Response to mTOR inhibitors in in-vitro experiments [30]
*IDH1*	p.R132H (c.G395A)	8	Response to IDH1 and pan-IDH inhibitors (active clinical trial) [31]
*MET*	Amplification	7	Response to c-MET inhibitors
*NF1*	c.A3975-2 T	6	Possibly increased sensitivity to MEK inhibitors [32]
*PIK3CA*	p.E545K (c.G1633A)	12	Response to PI3K/AKT/mTOR inhibitors [26, 27]
	p.M1043 V (c.A3127G)	2	
	Amplification	13, 7	
*PTEN*	p.R142fs (c.425delG)	6	Response to PI3K/AKT/mTOR inhibitors [27]
*TP53*	p.Y234C (c.A701G)	13	Better response to bevacizumab [24]; Better response to pazopanib in advanced sarcoma [25]
	p.R273C (c.C817T)	9	
	Nonsense	2, 7	
	Frameshift	2, 5, 12	

## Discussion

In this study, we analyzed the genomic profiles of 15 ESC patients by targeted sequencing of pan-cancer genes in archived primary FFPE tissue samples. For the first time, we obtained an overall picture of the genomic alterations for this rare disease that could be inspirational for developing and selecting targeted therapies in the future. Despite the differences of cellular morphology between sarcoma and carcinoma components in the tumor, they seem to share the majority of the mutations according to a study of uterine carcinosarcoma [4]. By comparing the mutational profiles of these ESC patients to a previous study on Chinese ESCC patients, we found similarities but also differences of genomic alterations between these two diseases. Typically, *TP53* was mutated in all ESC cases, which is comparable to 93% in ESCC [11]. Besides, histone modifier genes show a high mutation rate in both ESC (40%) and ESCC (63%), and *EP300* and its homolog *CREBBP* are the most commonly mutated genes. *EP300* mutations were associated with shorter overall survival (OS) time (median survival time around 20 months) in ESCC [11]. In the ESC patients we tested, the OS time of 3 cases with *EP300* alterations are > 10 months, > 12 months (both alive at the time of the return visit) and 27 months. A longer observation time is required to compare the survival rates of ESC patients with or without *EP300* mutations.

In addition, 5 out of 15 (33%) patients have *PIK3CA* alterations, much higher than what was observed in ESCC (5%) (*p* = 0.0002) [11]. All 5 cases with *PIK3CA* alterations were male patients at the stage I-II diseases with smoking history. The OS of these patients show tremendous differences, ranging from 1 to 47 months. Due to the limit sample size, we are not be able to link *PIK3CA* alterations to the possibility of early diagnosis or poor prognosis, but *PIK3CA* alterations are now clinically actionable with potential responses to PI3K/AKT/mTOR inhibitors [33, 34]. Patients would be subjected to targeted treatment and their responses and disease recurrence can be monitored by regular inspection of this alteration.

This study uncovered the diversity of tumor genetic background in ESC patients. Despite 5 patients carried mutations on MMR and proofreading genes, half of them were predicted to be benign. Therefore, it is not surprised that these 5 patients did not show a significant higher TMB comparing to other patients. The reason is possibly the functional overlapping of MMR proteins that could compensate the functional loss of each other [35], which is also explanatory in patient #2, as he had more than one MMR genes altered and his TMB level is relatively higher comparing to others.

## Conclusions

To our knowledge, this is the very first study specifically investigated the genomic alterations in ESC patients. Patients carrying these mutations could be potentially treated with targeted drugs to improve their long-term prognosis. The observations of high mutation rates in *PIK3CA* and histone modifier genes need to be validated in a larger sample size in the future if used to define clinical strategies for personalized therapy for this disease.

## Additional files

Additional file 1: **Table S1.** The gene list of the gene panel used for the targeted sequencing. It includes 416 cancer-relevant genes and introns of 16 fusions genes (bold text). (XLSX 13 kb)

Additional file 2: **Table S2.** Gene alterations in all patients and their functional and clinical significance annotation. All mutations were annotated with SIFT, CLINVAR and COSMIC in ANNOVAR software. (XLSX 21 kb)

## Abbreviations

ESC: Esophageal sarcomatoid carcinoma
ESCC: Esophageal squamous cell carcinoma
FFPE: Formalin-fixed paraffin-embedded
HMT: Histone methyltransferase complex
MAF: Mutation allele frequency
MMR: Mismatch repair
NGS: Next generation sequencing
RTK: Receptor tyrosine kinase
TMB: Tumor mutation burden
